# Association of single nucleotide polymorphisms in the *F2RL1* gene with clinical and inflammatory characteristics of patients with asthma

**DOI:** 10.1186/s13223-024-00873-z

**Published:** 2024-02-02

**Authors:** Nami Shrestha Palikhe, Qahir Haji, Emily Mack, Tristan Sinnatamby, Andrew J. Sandford, Lisa Cameron, Harissios Vliagoftis

**Affiliations:** 1 Division of Pulmonary Medicine, Department of Medicine, Faculty of Medicine & Dentistry, University of Alberta, 550 A HMRC, Edmonton, AB T6G 2S2 Canada; 2 Alberta Respiratory Centre, University of Alberta, Edmonton, AB Canada; 3 Centre for Heart Lung Innovation, St. Paul’s Hospital, University of British Columbia, Vancouver, BC Canada; 4 Department of Pathology and Laboratory Medicine, Schulich School of Medicine and Dentistry, Western University, London, ON Canada; 5 Present Address: Faculty of Education, University of Alberta, Edmonton, Canada

**Keywords:** Single nucleotide polymorphism, F2RL1 gene, Asthma

## Abstract

**Background:**

Proteinase-activated receptor 2 (PAR-2) is a G-protein coupled receptor associated with many inflammatory diseases, including asthma. We have shown an association between PAR-2 expression in peripheral blood monocytes and asthma severity as well as blood PAR-2 mRNA level and lung function. Since *F2RL1* (the gene encoding PAR-2) polymorphisms affect PAR-2 expression, we hypothesize they may affect asthma severity.

**Methods:**

We recruited 76 subjects with asthma of varying severity and collected clinical (FEV_1_ [% predicted], FEV_1_/FVC, IgE) and immunological (PAR-2 mRNA, blood eosinophils) disease parameters. We also genotyped these individuals for 3 *F2RL1* SNPs (-45C/T, -149C/G, c.621C/T).

**Results:**

We found that the *F2RL1* SNP “C” allele of -45C/T (rs1529505) was associated with PAR-2 mRNA and blood eosinophils. *F2RL1* SNP c.621C/T (rs631465) was associated with PAR-2 mRNA. The *F2RL1* SNP -149C/G (rs2242991) had no association with any of the parameters studied. This study identified one *F2RL1* SNP rs1529505 is associated with parameters of asthma, but not asthma severity.

**Conclusion:**

Larger studies are needed to further elucidate the role of PAR-2 in the pathophysiology of asthma and the influence of genetic variation.

## Background

Affecting approximately 3 million Canadians, asthma is a chronic respiratory disease usually characterized by eosinophilic airway inflammation and the presence of TH2 cytokines [1]. Most patients with allergic asthma exhibit TH2 inflammation including airway infiltration by eosinophils and CD4^+^ cells. With asthma prevalence continuing to rise, a better understanding of asthma pathophysiology is needed to inform novel asthma therapeutic strategies.

Proteinase-activated receptors (PARs) are a family of G-protein coupled receptors widely expressed throughout the body [2]. Of the four PARs identified to date (PAR-1 to PAR-4), PAR-2 has been associated with multiple inflammatory conditions, including asthma [3, 4]. Murine studies have demonstrated that PAR-2 activation is crucial for allergic sensitization, eosinophilic inflammation, airway remodeling and changes in airway physiology [3, 4]. In humans, PAR-2 expression is increased on airway epithelial cells [5] and smooth muscle cells [6] of patients with asthma, while PAR-2 expression on peripheral blood monocyte subsets correlates with asthma severity [7]. PAR-2 is encoded by the coagulation factor II receptor-like 1(*F2RL1*) gene on chromosome 5 [8]. *F2RL1* single nucleotide polymorphisms (SNPs) have been associated with inflammatory conditions including atopy (rs631465), osteoarthritis (rs1529505 and rs2242991) and obesity (rs631465) [9–11]. However, there are no studies showing associations between genetic variation within the *F2RL1* gene and asthma characteristics.

To date, 27 SNPs have been shown to influence *F2RL1* expression in monocytes [12], monocytes being the main cell expressing PAR-2 in the blood. The rs1529505 SNP genotyped in this study was linked with most of these SNPs. We also genotyped another two SNPs, rs2242991 and rs631465, which have low linkage disequilibrium with rs1529505 but have an association with asthma [9]. In the 1000Genomes data [13], the linkage disequilibrium (LD) between rs1529505 and rs2242991 is r^2^ < 0.30 and between rs1529505 and rs631465 it is r^2^ < 0.39. We hypothesized that *F2RL1* SNPs associated with increased PAR-2 expression would also be associated with increased inflammation and consequently asthma severity.

## Methods

### Subjects

From March 2012 to November 2013 [7] and April 2015 to December 2017 [14] seventy-six adult patients > 18 years of age with a clinical diagnosis of asthma (confirmed by FEV1 reversibility ≥ 12% or 200 ml increase in FEV1) were recruited from the University of Alberta Asthma Clinics after giving informed consent. Approval for this study was obtained from the University of Alberta Research Ethics Board (Pro00001784). We have previously published data obtained from subgroups of this cohort [7, 14].

Demographic characteristics (age, gender, body mass index, history of smoking, current smoking status), peripheral blood eosinophil counts and pulmonary function data (% predicted FEV_1_ and FEV_1_/FVC)- values calculated from the Global Lung Initiative reference set [15], as well as information about usage of steroids and biologics were recorded from electronic patient records at the time of recruitment. Asthma severity was determined using American Thoracic Society (ATS) guidelines [16]. Peripheral venous blood samples were collected at the time of recruitment.

### *F2RL1* genotyping

**Fig. 1:**
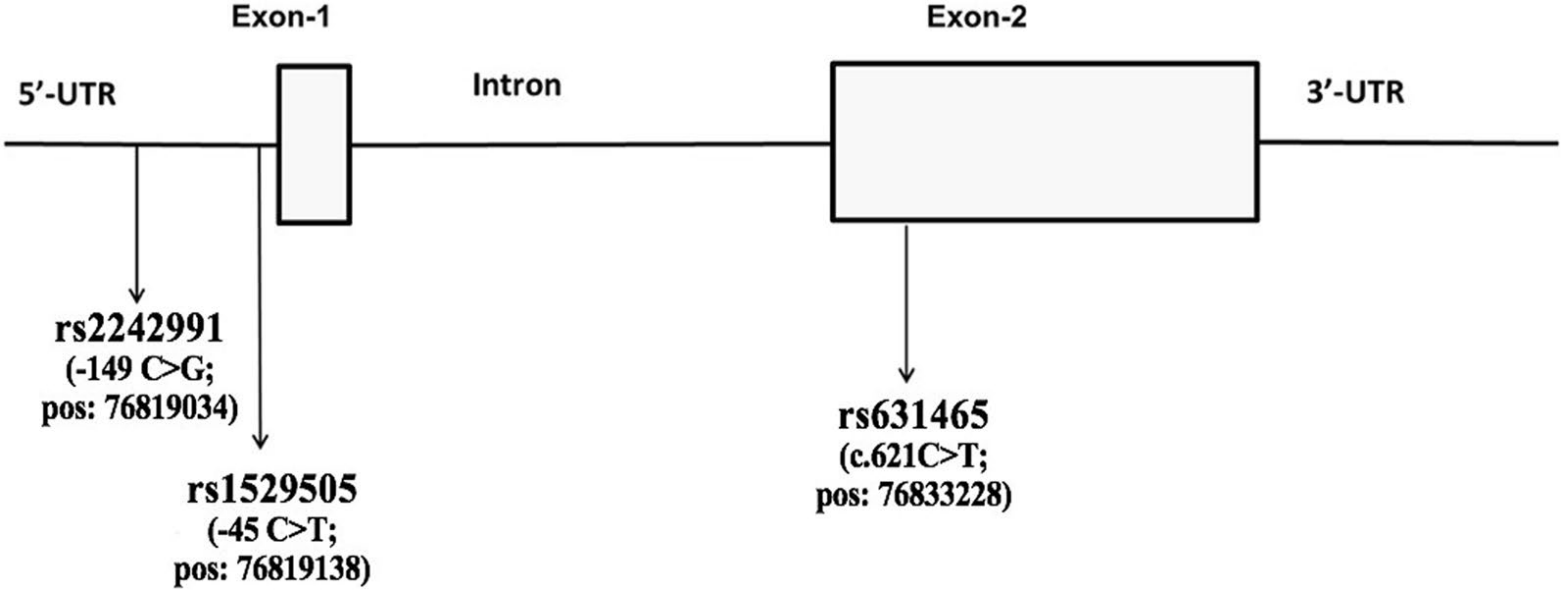
The *F2RL1* gene (5q13.3), with the positions of the three investigated SNPs (rs1529505, rs631465 and rs2242991)

*F2RL1* gene structure and SNPs positions are shown in Fig. 1*. F2RL1* TaqMan SNP genotyping assays (Life Technologies, MA, USA) were used to genotype the three SNPs (rs1529505, rs631465 and rs2242991) in genomic DNA (gDNA) extracted from peripheral venous blood.

### Quantitative real time PCR (qRT-PCR)

Whole blood RNA was extracted from peripheral blood collected in PAXgene whole blood RNA tubes (PreAnalytiX, Qiagen/BD) using the PAXgene Blood RNA kit (PreAnalytiX). PAR-2 mRNA expression was quantified with a quantitative reverse transcription PCR (RT-qPCR) assay we described [7]. GAPDH mRNA was used as housekeeping control and was quantified using a custom 6FAM-labeled TAMRA probe. The PCR protocol consisted of 10 min at 95 ℃ followed by 40 cycles of 30 s at 95 ℃ and 60 s at 60 ℃. Data were calculated and represented as “PAR-2 copies per 1000 copies of GAPDH”.

### Peripheral blood flow cytometry

Whole blood flow cytometry for PAR-2 expression on monocyte subsets and for the presence of TH2 cells was performed as we described [7, 17]. Monocyte subsets were defined as CD14^++^CD16^−^ (classical) and CD14^++^CD16^+^ (intermediate) within the lymphocyte/monocyte gate of side scatter/forward scatter plots. TH2 cells were identified as CRTh2-expressing CD4^+^ T cells. Flow cytometry was performed using an LSR II flow cytometer. Results were analyzed by FlowJo^®^.

### Enzyme-linked immunosorbent assay (ELISA)

Commercially available ELISA kits for IL-13 (Interleukin-13,), PGD2 (Prostaglandin D2; MOX ELISA kit, Cayman) and CCL5 (R and D) were used.

### Statistical analysis

The dominant and additive model was used for SNP analysis. The Mann–Whitney U test was used to analyze correlations between *F2RL1* SNP genotypes and all continuous variables (PAR-2 mRNA expression, PAR-2 expression on monocytes, eosinophil and TH2 cell counts, serum CCL5 and IL-13 concentrations, % predicted FEV_1_ and FEV_1_/FVC) (Table 1). The association between SNPs genotypes and asthma severity was analyzed using the Chi-square test. SPSS 28.0 software was used to conduct all statistical analysis. *P *values of < 0.05 were considered significant.

**Table 1:** Demographic characteristics of study cohorts

Parameters	Total (n = 76)	Mild/Moderate (n = 53)	Severe (n = 23)	Mild/Moderate Vs Severe (*p*-value)
Age (y) (median [IQR])	39 (30–52)	38 (29–50)	42 (30–57)	0.381
Sex-Female (n [%])	46 (60.5%)	33 (62.3%)	13 (56.5%)	0.799
BMI (kg/m^2^) (median [IQR])	27.8 (23.1–30.9)	27.9 (23.0–33.8)	27.0 (23.3–28.7)	0.354
Ethnicity (Caucasian) (n [%})	65 (85.5%)	45 (84.9%)	20 (87.0%)	1.000
History of smoking (n[%})	25 (32.9%)	15 (28.3%)	10 (43.5%)	0.288
Current smoking (n[%])	5 (6.6%)	4 (7.5%)	1 (4.3%)	1.000
FEV1 (% predicted) (median [IQR])	86 (71.0–92.7)	90 (79–97.5)	65 (53–89)	** < 0.001**
FEV1/FVC (median [IQR])	82.5 (69.0–87.7)	84.0 (73.5–91.0)	72.0 (57–83)	** < 0.001**
IgE* (kU/L) (median [IQR])	1.78 (1.28–2.20)	1.79 (1.36–2.23)	1.72 (1.17–2.15)	0.632
CCL5 (ng/ml)	50 (36.3–77.6)	42.7 (34.0–64.1)	71.4 (42.1–83.5)	**0.018**
PGD2 (pg/ml)	346 (130–769)	344 (115–848)	388 (212–765)	0.665
IL-13 (pg/ml)	4.47 (1–22.3)	2.1 (1.0–14.2)	15.0 (1–42.7)	0.051
Total ICS dose (fluticasone equivalent-µg/day)	393 (0–1000)	137 (0–500)	1000 (1000–1173)	** < 0.001**
Biologics (n[%])	6 (7.8%)	0 (0%)	6 (26%)	** < 0.001**

Bold value denote statistical significance at the p<0.05

IgE*; Mild/Moderate = 31, Severe = 22, CCL5; Mild/Moderate = 52, Severe = 23, IL-13; Mild/Moderate = 41, Severe = 21, PGD2; Mild/Moderate = 34, Severe = 21, because of missing data points

*BMI* Body mass index, *FEV1* Forced expiratory volume in 1 s, *FVC* Forced vital capacity, *Ig* immunoglobulin, *IQR* interquartile range, *y* year, *CCL5* Chemokine ligand 5, *PGD2* Prostaglandin D2, *IL-13* Interleukins 13

## Results

### Genotype and allele frequencies

Our full cohort consists of 76 subjects, 53 with mild/moderate asthma and 23 with severe asthma; the majority of our population was Caucasian (n = 62). Demographics are shown in Table 1. The severe asthma group had significantly lower FEV1 (% predicted) compared to mild/moderate asthma (p < 0.001) while there were no differences in age, sex, body mass index (BMI), IgE, status of smoking between the two groups. Subjects in the severe asthma group had significantly higher inhaled corticosteroid (ICS) use and more subjects on biologics compared to subjects in the mild/moderate asthma group (p < 0.001). Table 2 shows the genotype and allele frequencies for the rs1529505, rs2242991 and rs631465 SNPs in the study cohort and in subjects with mild/moderate and severe asthma separately. The genotype distribution for all three SNPs was not significantly different from what would be expected based on Hardy–Weinberg equilibrium (p > 0.05). The minor allele frequencies (MAFs) in the cohort were comparable to both global MAFs and MAFs calculated in the 1000 Genomes Project for admixed American and European cohorts.

**Table 2:** Genotype and Allele Frequency of *F2RL1* Polymorphisms in subjects

F2RL1 SNPs	Genotype or Allele	Total (N = 76)	Mild/Moderate	Severe
F2RL1	CC	24 (31.6%)	17 (32.1%)	7 (30.4%)
-45C/T	CT	31 (40.8%)	20 (37.7%)	11 (47.8%)
(rs1529505)	TT	21 (27.6%)	16 (30.2%)	5 (21.7%)
	MAF	0.48	0.49	0.45
F2RL1	CC	60 (81.1%)	43 (82.7%)	17 (77.3%)
-149C/G^*^	CG	14 (18.9%)	9 (17.3%)	5 (22.7%)
(rs2242991)	GG	0 (0%)	0 (0%)	0 (0%)
	MAF	0.10	0.09	0.11
F2RL1	CC	68 (89.5%)	46 (86.8%)	22 (95.7%)
c.621C/T	CT	8 (10.5%)	7 (13.2%)	1 (4.3%)
(rs631465)	TT	0 (0%)	0 (0%)	0 (0%)
	MAF	0.05	0.06	0.02

^*^n = 74, because of missing data points

### *F2RL1* rs1529505 genotype and asthma

The CC genotypes of *F2RL1* rs1529505 were not associated with clinical characteristics of asthma in both the additive and the dominant model; there was no association with asthma severity (p > 0.05, Table 2), FEV_1_ (% predicted), FEV_1_/FVC or serum IgE levels (p > 0.05, Table 3). No significant difference was observed between individuals with the CT and TT genotypes of the rs1529505 (p > 0.05).

**Table 3:** Comparison of the clinical characteristics according to the genotype of *F2RL1* -45C/T

	CC (n = 24)	CT (n = 31)	TT (n = 21)	CC vs CT + TT *p*-value
Age (y) (median [IQR])	36 (27–49)	47 (30–55)	37 (32–52)	0.234
Sex-Female (n [%])	13 (54.2%)	22 (71.0%)	11 (52.4%)	0.460
BMI (kg/m2) (median [IQR])	28.7(23.7–34.1)	27.7 (24.1–30.5)	25.8 (21.2–38.3)	0.241
History of smoking (n[%])	10 (41.7%)	8 (25.8%)	7 (33.3%)	0.302
FEV_1_ (% predicted) (median [IQR])	88.5 (76–91.7)	80.0 (67.0–91.0)	91.0 (77.5–102.5)	0.806
FEV_1_/FVC(% predicted) (median [IQR])	83.0 (78.0–90.2)	73.0 (65.0–86.0)	84.0 (73.5–94.0)	0.196
IgE* (kU/L) (median [IQR])	1.73 (1.1–2.0)	1.68 (1.39–2.19)	1.99 (1.31–2.55)	0.304
Eosinophils** (cells/µl)	200 (100–300)	300 (200–500)	305 (200–575)	**0.029**
Proportion of TH2 cells/WBC^#^)	0.188(0.135–0.352)	0.37 (0.18–0.54)	0.24 (0.19–0.44)	**0.013**
PAR-2 mRNA^##^Expression (copies/1000 GAPDH copies)	26.91 (14.2–41.7)	15.97(8.01–23.66)	14.87(4.50–22.57)	**0.014**
Percentage of IMMo expressing PAR-2^&^	11.4 (7.20–29.3)	13.3 (5.19–38.0)	16.5 (6.15–32.57)	0.891
Percentage of CMo expressing PAR-2^&&^	10.7 (2.20–15.80)	5.40 (1.50–9.63)	5.45 (1.85–15.80)	0.391
CCL5 (ng/ml)^¶^	42.8 (27.1–74.63)	50.57 (37.32–80.13)	47.4 (33.4–71.32)	0.323
PGD2 (pg/ml)^∞^	449.94(106.79–769.38)	322.04 (132.56–792.81)	364.89 (165.88–897.17)	0.710
IL-13 (pg/ml)^€^	9.1 (1.0–21.32)	2.84 (1.0–36.6)	1.85 (1.0–19.19)	0.521

Bold value denote statistical significance at the p<0.05

^*^n = CC = 15, CT = 22, TT = 16

^**^n = CC = 23, CT = 30, TT = 20

^#^n = CC = 24, CT = 31, TT = 19

^##^n = CC = 17, CT = 23, TT = 12

^&^n and ^&&^n = CC = 15, CT = 23, TT = 12

^¶^n = CC = 24, CT = 30, TT = 21

^∞^n = CC = 19, CT = 20, TT = 16

^€^n = CC = 21, CT = 23, TT = 18

**Fig. 2:**
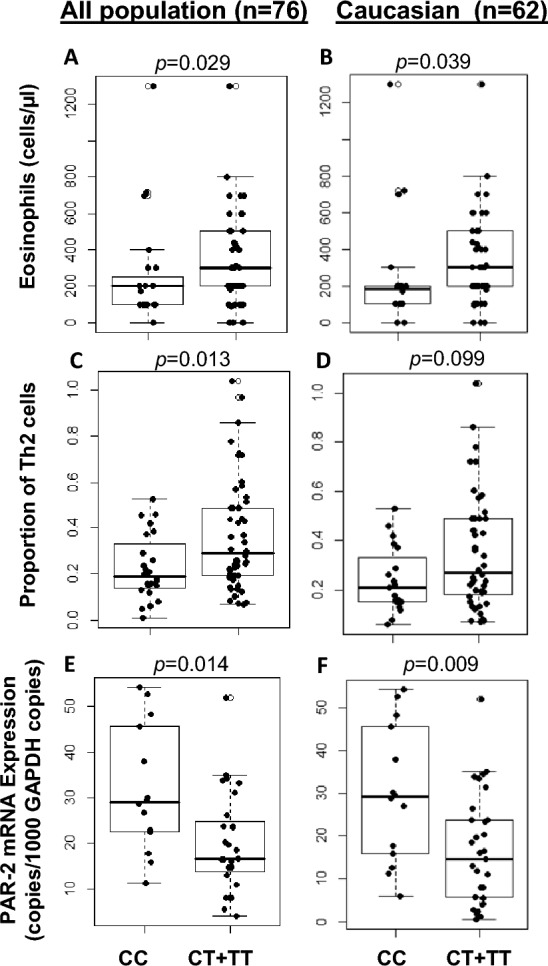
**A**. Differences in median eosinophil counts between rs1529505 SNP genotypes in the whole study cohort and **B**. Caucasian. Eosinophil counts were obtained from complete blood counts in the patient charts. **C**. Differences in the median % of TH2 cells in peripheral blood in whole study cohort and **D**. Caucasian. Measurements for % of TH2 cells were performed by flow cytometry. **E**. Differences in median whole blood PAR-2 mRNA expression between rs1529505 SNP genotypes in the whole study cohort and **F**. Caucasian. The whole blood PAR-2 mRNA expression was measured using qRT-PCR

Individuals with the CT and TT genotypes for the rs1529505 SNP had significantly increased peripheral blood eosinophil counts compared to individuals with the CC genotype (p = 0.029, Fig. 2A); this finding was also evident for the Caucasian population of our cohort (n = 62, p = 0.039, Fig. 2B). Individuals with the CT and TT genotypes for the rs1529505 SNP also had an increased percentage of TH2 cells in peripheral blood relative to individuals with the CC genotype (p = 0.013, Fig. 2C). However, this observation was not statistically significant for the Caucasian population of our cohort (n = 62, p = 0.099), Fig. 2D).

In contrast to what was shown for inflammatory cell counts, individuals with the CT and TT genotype for the rs1529505 SNP had decreased whole blood PAR-2 mRNA expression relative to individuals with the CC genotype in both whole (p = 0.014) and Caucasian population (p = 0.009) in our cohort study (Fig. 2E, F). The rs1529505 SNP was not associated with PAR-2 surface expression on blood classical or intermediate monocytes (Table 3).

Since we are interested to understand the regulation of T2 inflammation in asthma, we studied if *F2RL1* rs1529505 is associated with TH2 cells, TH2 cells’ ligand PGD2, cytokines IL-13 and CCL5, a T cell chemoattractant. The rs1529505 genotypes had no association with PGD2, IL-13 or CCL5 levels in serum (Table 3). There were no significant differences in blood eosinophils, PAR-2 mRNA expression and percentage of TH2 cells when we compared between CC vs CT vs TT (p > 0.05, Table 3).

**Fig. 3:**
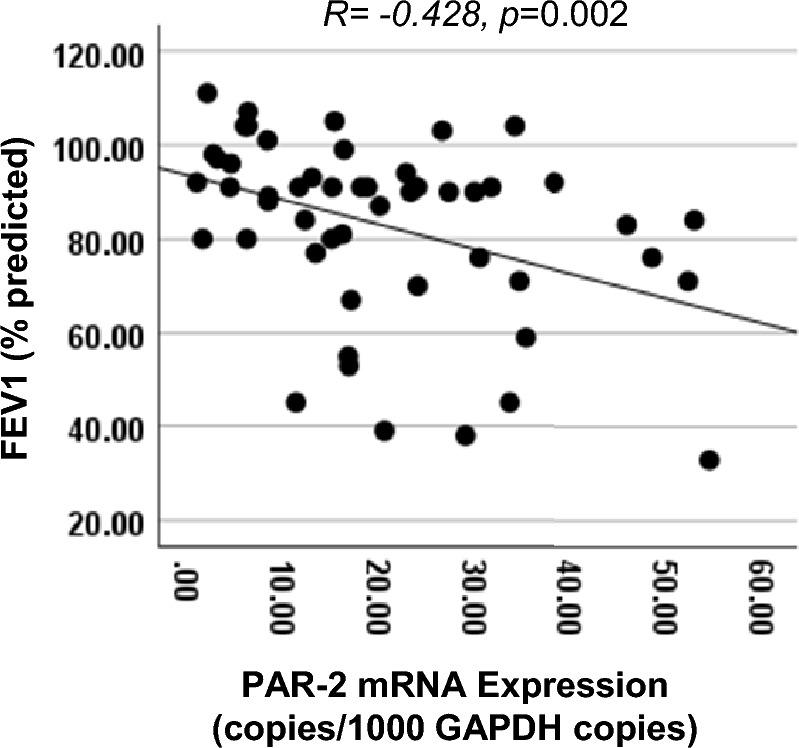
The correlation between whole blood PAR-2 mRNA expression and FEV1 (% predicted) in the whole study cohort

There was a negative correlation of whole blood PAR-2 mRNA with FEV1 (% predicted) in our current cohort (R = − 0.428, p = 0.002, n = 52) (Fig. 3), similar to the result we reported for a smaller cohort [7].

### *F2RL1* rs2242991 and rs631465 genotypes and asthma

There was no association of the rs2242991 genotypes with any of the clinical and immunological markers described above (Table 4). Individuals with CC genotype of rs631465 SNP had higher PAR-2 mRNA compared to individuals with CT genotype (p = 0.018; Table 5); there were no subjects with the TT genotype in our cohort. Interestingly, individuals with CC genotype of rs631465 SNP had a trend towards higher BMI compared to individuals with CT genotype (p = 0.055; Table 5), in agreement with a previous observation [10].

**Table 4:** Comparison of the clinical and immunological characteristics according to the genotype of *F2RL1* -149C/G

	CC (n = 60)	CG (n = 14)	*p* value CC vs CG
Age (y) (median [IQR])	38.5 (30–51.7)	42.0 (28–53)	0.956
Sex-Female (n[%])	34 (56.7%)	11 (78.6%)	0.223
BMI (kg/m2) (median [IQR])	26.9(22.9–30.7)	28.3 (24.5–38.4)	0.174
History of smoking (n[%])	15 (25%)	9 (64.3%)	**0.009**
FEV_1_ (% predicted) (median [IQR])	85.5 (68–92.7)	90-(75–95)	0.624
FEV_1_/FVC (% predicted) (median [IQR])	83.0 (67.2–88.7)	79(70.7–8420)	0.369
IgE* (kU/L) (median [IQR])	1.71 (1.1–2.2)	2.03 (1.56–2.21)	0.261
Eosinophils** (cells/µl)	250 (177–500)	200 (100–370)	0.362
Proportion of TH2 cells/WBC^#^	0.24 (0.15–0.43)	0.32 (0.16–0.49)	0.527
PAR-2 mRNA^##^Expression (copies/1000 GAPDH copies)	15.7 (7.0–26.5)	26.61 (19.6–34.8)	0.088
Percentage of IMMo expressing PAR-2^&^	15.4 (6.68–32.35)	8.7 (6.92–29.30)	0.715
Percentage of CMo expressing PAR-2^&&^	5.5 (1.72–14.95)	7.7 (1.50–12.20)	0.804
CCL5 (ng/ml)^¶^	50.0 (37.26–77.68)	44.12 (26.85–69.57)	0.433
PGD2 (pg/ml)^∞^	388.45 (157.30–868.37)	285.55 (71.28–485.26)	0.102
IL-13 (pg/ml)^€^	4.75 (1.0–19.10)	1.0 (1.0–22.77)	0.520

Bold value denote statistical significance at the p<0.05

^*^n = CC = 42, CG = 9

^**^n = CC = 58, CG = 13

^#^n = CC = 58, CG = 14

^##^n = CC = 41, CG = 10

^&^n and ^&&^n = CC = 41, CG = 7

^¶^n = CC = 59, CG = 14

^∞^n = CC = 19, CG = 12

^€^n = CC = 21, CG = 13

**Table 5:** Comparison of the clinical and immunological characteristics according to the genotype of F2RL1 c.621C/T

	CC (n = 68)	CT (n = 8)	*p* value CC vs CT
Age (y) (median [IQR])	39 (30–53)	34.5 (19.5–43.7)	1.000
Sex-Female (n[%])	44 (64.7%)	2 (25%)	0.052
BMI (kg/m2) (median [IQR])	27.9(23.3–31.5)	23.3 (21.3–27.4)	0.055
History of smoking (n[%])	24 (35.3%)	1 (12.5%)	0**.**259
FEV_1_ (% predicted) (median [IQR])	85.5 (70.2–92.0)	89-(80.2–102.2)	0.301
FEV_1_/FVC(% predicted) (median [IQR])	82.0 (69–87.7)	83.5(65.5–89.7)	0.865
IgE* (kU/L) (median [IQR])	1.73 (1.2–2.2)	1.86 (1.79-)	0.889
Eosinophils** (cells/µl)	200 (100–440)	250 (172–705)	0.618
Proportion of TH2 cells/WBC^#^	0.24 (0.15–0.46)	0.25 (0.08–0.42)	0.470
PAR-2 mRNA^##^Expression (copies/1000 GAPDH copies)	19.7 (11.15–30.7)	6.0 (2.83–15.7)	**0.018**
Percentage of IMMo expressing PAR-2^&^	14.2 (6.03–31.0)	14.50 (7.76–47.27)	0.475
Percentage of CMo expressing PAR-2^&&^	5.45 (1.37–12.05)	14.95 (5.08–28.07)	**0.053**
CCL5 (ng/ml)^¶^	50.95 (36.59–78.15)	41.53 (24.14–52.73)	0.198
PGD2 (pg/ml)^∞^	346.93 (130.7–769.38)	492.54 (148.57–1809.07)	0.674
IL-13 (pg/ml)^€^	3.52 (1.0–33.39)	8.67 (1.32–43.75)	1.000

Bold value denote statistical significance at the p<0.05

^*^n = CC = 51, CG = 2

^**^n = CC = 67, CG = 6

^#^n = CC = 66, CG = 8

^##^n = CC = 45, CG = 7

^&^n and ^&&^n = CC = 42, CT = 8

^¶^n = CC = 67, CG = 14

^∞^n = CC = 51, CG = 12

^€^n = CC = 54, CG = 13

## Discussion

PAR-2 plays a prominent role in airway inflammation and asthma [5, 7, 18]. This is the first study exploring associations between *F2RL1* gene SNPs and clinical and immunological characteristics of asthma. We genotyped three SNPs (rs1529505, rs2242991 and rs631465) in *F2RL1* gene since they are not linked to each other, and previous studies have shown that these three SNPs are associated with monocytes [12] and asthma [9]. We had hypothesized that *F2RL1* SNP genotypes that increase whole blood PAR-2 expression would be associated with increased inflammatory markers in blood and increased asthma severity, but the data do not support this hypothesis. In fact, rs1529505 (CT and TT), was associated with a higher frequency of eosinophils and TH2 cells), but decreased PAR-2 mRNA expression in peripheral blood. In addition, the genotype had no association with disease severity or lung function. These results suggest that the relationship between PAR-2 expression and characteristics of asthma is quite complex. On the other hand, the CC genotype of rs631465 was associated with decreased PAR-2 mRNA expression, but rs2242991 was not associated with any of the parameters we analyzed here.

Our observation that carries of the heterozygote and minor rs1529505 genotype (CT and TT) had less PAR-2 mRNA in peripheral blood is concordant with data on whole blood and lung found in the Genotype-Tissue Expression (GTEx) portal. However, this association is not consistent across all tissues. For example, in other tissues the opposite has been observed; the major genotype rs1529505 CC is associated with reduced PAR-2 in the gastrocnemius muscle (GTEx) and in synovial tissue (11). Based on in silico prediction software (AliBaba2.1), the rs1529505 SNP “CC” genotype may affect binding sites for Sp1 and AP-2alpha transcription factors, emphasizing a potential role for this SNP in modulating PAR-2 mRNA expression. Therefore, differential association of the CC genotype with PAR-2 mRNA expression in various tissues may be the result of differential expression of transcription factors across tissues or across the dominant PAR-2-expressing cell in peripheral blood and/or certain tissue. Both Sp1 [19, 20] and AP-2alpha [21, 22] have been associated with asthma and regulation of asthma related factors.

While the rs1529505 SNP was associated with whole blood PAR-2 mRNA expression in our cohort, there was no association between this SNP and surface PAR-2 protein expression on peripheral blood monocytes. However, a large proportion of PAR-2 expressed by monocytes is intracellular [23], which indicates that surface PAR-2 expression on monocytes may have little to do with mRNA abundance. Alternatively, the significant association observed between the CC genotype of rs1529505 and increased PAR-2 mRNA expression in whole blood may be the result of increased PAR-2 mRNA expression in other blood PAR-2-expressing cells, such as eosinophils, T lymphocytes or dendritic cells [24–27].

One reason for the seemingly opposite finding of rs1529505 CC genotype with increased PAR-2 expression, yet lower blood eosinophil and TH2 cell counts, could be enhanced infiltration to the lung. Indeed, PAR-2 expression in the airways promotes eosinophil infiltration in murine models [28]. If the CC genotype of rs1529505 also increases PAR-2 expression in the lungs, it may lead to increased inflammatory cell migration from peripheral blood into the airways resulting in decreased numbers of eosinophils and TH2 cells circulating in the blood. Future studies should explore associations between the rs1529505 SNP, airway PAR-2 expression and airway eosinophil counts to test this hypothesis.

The rs1529505 SNP was not associated with asthma severity in this study, nor with any other clinical characteristic, or serum cytokine levels. The reason for this lack of association despite higher numbers of eosinophils and TH2 cells in peripheral blood, is not clear. Larger cohort studies and analysis of more immune and clinical markers associated with asthma severity may clarify this point. We also replicated in our current cohort our previous observation in a smaller cohort [7], that there is a negative correlation between whole blood PAR-2 mRNA and FEV1 (% predicted).

The CC genotype of rs631465 SNP was associated with increased PAR-2 mRNA expression compared to CT genotype in this study cohort. However, this finding is contradictory to a previously published observation in Korean children where the expression of PAR-2 mRNA in PBMC of subjects with CT or TT genotype was higher than in those with the CC genotype of rs631465 [9]. This may be the result of the different ethnic backgrounds of our population.

The rs2242991 was not associated with any parameters studied. It is however interesting that even though we had a small number of individuals with CT genotype we identified a trend for association of the rs631465 SNPs with obesity, as has been shown before [10]. Ultimately, larger studies are needed to explore the association of the rs2242991 and rs631465 SNPs with asthma, which might also depend on the ethnicity of the individuals in the studied cohort.

Our study has several limitations that need to be considered. We have a small sample size, and this may limit our ability to identify small differences between genotypes for other biomarkers. We also did not recruit normal controls to compare with our asthmatic population, since our aim was to test whether PAR-2 SNPs are associated with asthma severity. However, for the other parameters we analyzed, the addition of a normal cohort may have solidified our results.

## Conclusion

This study identified one *F2RL1* SNP that is associated with various parameters of asthma, but not asthma severity. Larger studies are needed to further elucidate the role of PAR-2 in the pathophysiology of asthma and the influence of genetic variation.

## Data Availability

The datasets used and/or analyzed during the current study are available from the corresponding author on reasonable request.
